# New insights into HTLV entry

**DOI:** 10.1186/1742-4690-8-S1-A183

**Published:** 2011-06-06

**Authors:** Claudine Pique, Olivier Hermine, Francis W Ruscetti, Kathryn S Jones

**Affiliations:** 1 INSERM, U1016, Institut Cochinn, Paris, France; 2 CNRS UMR 8104, Paris, France; 3 Univ Paris Descartes, Paris, France; 4 Service d’Hématologie Adulte, Hôpital Necker, Paris, CEDEX 15, France; 5 Cancer and Inflammation Program, NCI-Frederick, Frederick, Maryland, 21702, USA; 6 Basic Research Program, SAIC-Frederick, Inc., Frederick, Maryland, 21702, USA

## 

Insight into the long-lasting mystery of the HTLV-1 entry receptor was provided over the past several years by the independent identification of three molecules required for binding and entry into cells: heparan sulfate proteoglycans (HSPG), the VEGF-165 receptor Neuropilin 1 (NRP-1) and the ubiquitous glucose transporter GLUT1. Further studies have indicated that these three molecules work together to ensure virus binding and ultimately, fusion of the viral and target cell membranes. Interaction of the virus with this receptor complex is mediated by the envelope glycoproteins (Env), which consists of a complex formed between a surface (SU, gp46) and a transmembrane (TM, gp21) subunit. Studies prior to the characterization of the HTLV-1 receptor complex identified domains and specific residues of the HTLV-1 SU involved in the Env/receptor interactions through either characterization of neutralizing antibodies or peptides or analysis of Env mutants. We will present our current model for HTLV-1 entry and revisit the data about the functional domains of the SU in regard to the recently acquired knowledge about the three receptor molecules. We will also discuss the role of these three molecules during the cell-cell and cell-free infection of T cells and dendritic cells. Finally, we will examine the similarities and differences between the distinct members of the HTLV family in their receptor usage.

